# Comparative Analysis of Food Addiction and Obesity: A Critical Review

**DOI:** 10.1002/fsn3.70799

**Published:** 2025-08-15

**Authors:** Sammra Maqsood, Faiyaz Ahmed, Muhammad Tayyab Arshad, Ali Ikram, Muhammed Adem Abdullahi

**Affiliations:** ^1^ National Institute of Food Science and Technology University of Agriculture Faisalabad Faisalabad Pakistan; ^2^ Department of Basic Health Sciences, College of Applied Medical Sciences Qassim University Buraydah Saudi Arabia; ^3^ Functional Food and Nutrition Program, Faculty of Agro‐Industry Prince of Songkla University Hatyai Songkhla Thailand; ^4^ University Institute of Food Science and Technology The University of Lahore Lahore Pakistan; ^5^ Department of Food Science and Postharvest Technology Jimma University College of Agriculture and Veterinary Medicine, Jimma University Jimma Ethiopia

**Keywords:** food addiction, highly palatable foods, nutritional interventions, obesity

## Abstract

Compulsive ingestion of tasty foods is a characteristic of food addiction (FA), a debated but increasingly recognized disorder that shares neurological features with drug‐use disorders. This review examines the overlapping processes between FA and obesity, with a focus on the opioid and dopamine reward systems. Sweet, fatty, and salty processed foods dominate our brain pathways, making them increasingly less able to feel full and urge us to eat more than we require. While the diagnosis remains controversial, the Yale Food Addiction Scale (YFAS) is the most widely used assessment instrument. Potential management options include nutrition‐based interventions, such as whole‐food diets, and behavioral strategies, such as cognitive‐behavioral therapy (CBT) and conscious eating. However, stigmatization, labeling, and food industry promotion of addictive products pose ethical concerns. Against the backdrop of ongoing controversy, this article summarizes the highest‐quality research available on the neurological basis of FA as well as its measurement challenges and possible therapeutic options. The public health implications of FA and obesity can be lessened by future research to explore personalized treatments, refine diagnostic systems, and inform policy.

## Graphical abstract

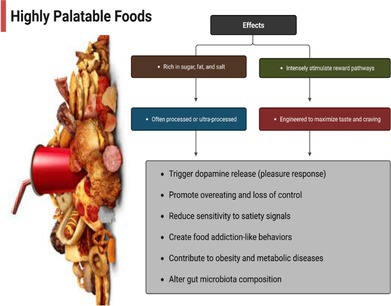

## 1 Introduction

Globally, the burden of obesity has tripled since 1975, and it is among the most significant public health issues of this century (Reilly et al. 2018). More than 650 million individuals worldwide are obese (BMI ≥ 30 kg/m^2^), with an additional 1.9 billion being overweight, as per the latest estimates (Blüher 2019). The epidemic is not confined to affluent countries; rather, it is spreading rapidly, particularly in urban cities across low‐ and middle‐income nations (Jaacks et al. 2019).

The huge health and economic burden is due to the fact that obesity is linked with a higher risk of type 2 diabetes, cardiovascular disease, cancer, and NAFLD (Ye et al. 2020; Quek et al. 2023). Seidell and Halberstadt (2015) identified several factors that lead to obesity. These include sedentary lifestyles, socioeconomic disparities, and excessive availability of energy‐dense, poor‐quality meals (Tufail et al. 2025). The growing rate of childhood obesity, which can cause metabolic problems over a lifetime, indicates that the disease is not confined to adults alone (Alfaris et al. 2023).

A condition referred to as sarcopenic obesity, where there is a simultaneous gain in fat and loss of muscle, is on the rise. It aggravates morbidity and affects older people more than anything else (Gao et al. 2021). Despite an increase in awareness among the populace of the condition (Blüher 2019), high rates of weight regain following typical weight loss therapies such as caloric cutting and exercise have continued. Therefore, researchers have considered alternative theoretical frameworks such as food addiction (FA) to predict compulsive eating. Other individuals may also experience neurobiological dysregulation, in line with substance dependence, as the underlying cause of obesity (Carter et al. 2016).

By refocusing our perspective on the brain mechanisms underlying health‐disordered eating, this position stands to drastically transform our approach to treating obesity. As explained by Gearhardt and Hebebrand (2021), some individuals exhibit the same behavioral and neurological response to highly attractive foods as individuals with substance use disorders, referred to as food addiction (FA). Evidence of shared brain links between drug addiction and compulsive consumption has offered empirical proof of FA; despite not being included in the DSM‐5 (Lindgren et al. 2018).

Research has demonstrated that 5%–20% of the general population and as many as 50% of those who are obese experience food addiction, based on the Yale Food Addiction Scale (YFAS), created in 2009 and remaining the most employed measurement for assessing FA symptoms (Adams et al. 2019). Calling overeating an “addiction” may medicalize normal behaviors or get individuals off the hook for what they do, critics say (Florio et al. 2022). However, neuroimaging research has indicated fascinating parallels in this regard. Both addiction and reduced prefrontal cortex activity, for instance, are marked by reduced dopamine D2 receptor availability in the reward pathways (Schulte et al. 2016).

Lennerz and Lennerz (2018) reported that animal models demonstrated that sugar and fat were as effective as addictive drugs in inducing neuroplastic changes. These findings suggest that some meals have addictive potential for vulnerable individuals. There are significant implications for clinical practice in debates on FA. Should it be affirmed as a distinct illness, it may warrant more targeted behavior and pharmaceutical intervention (Schulte et al. 2021). However, there are still impediments to FA diagnosis, particularly when attempting to distinguish FA from binge eating disorder (BED) and other forms of eating disorders (Piccinni et al. 2021). The role of the food industry in creating hyperpalatable products that circumvent the body's normal signals of fullness also generates further ethical issues (Wiss et al. 2020).

The mesolimbic dopamine system is involved in obesity and drug addiction, as shown by recent neuroscience research (Sinha 2018). The nucleus accumbens and orbitofrontal cortex, key components of the brain reward system, are more active in FA patients than in controls when presented with cues for palatable food, as functional magnetic resonance imaging research has demonstrated (Morales 2022). A state of tolerance is achieved when greater amounts are needed to produce the same pleasurable effect following the chronic intake of sugar and fat, which downregulates dopamine receptors (Lee and Dixon 2017).

The second essential aspect is the opioid system, which is responsible for controlling the hedonic (“liking”) aspects of food intake through endogenous opioids (Soroceanu et al. 2023). According to de Ceglia et al. (2021), individuals with FA often have cravings and insufficient control over particular foods even if they feel physically full. Gupta et al. (2020) indicated that the gut microbiota influences desires by immunological signaling and neurotransmitter production, and new studies have highlighted the gut–brain axis as a critical controller of eating behavior. As individuals become less sensitive to rewards, they tend to attempt to compensate for this with increased consumption of these foods, further worsening their metabolic dysfunction (Criscitelli and Avena 2016). Importantly, according to Sinha (2018), stress appears to exacerbate this process by activating the hypothalamic–pituitary–adrenal (HPA) axis, which subsequently heightens cravings for high‐calorie meals. Individuals who have suffered trauma are more likely to be obese and have FA, which could be due to the stress–reward relationship (Wiss et al. 2020).

This review discusses the neurobiology of food addiction (FA) and obesity, highlighting the similarities and disparities between FA and substance use disorders. This study aimed to elucidate the role of highly palatable meals and reward circuits (opioid systems and dopamine) in compulsive food consumption. In addition, it examines the challenges of defining FA as a distinct condition and evaluates current diagnostic tools, such as the Yale Food Addiction Scale (YFAS). Concerns regarding the consequences of food industry practices and FA classification ethics are secondary objectives and investigations into evidence‐based treatments such as whole‐food diets and behavioral interventions such as CBT and mindful eating. The objective of this review was to assist public health policy and clinical practice in combating FA and obesity by incorporating the most current findings.

## 2 Neurobiology of Food Addiction

The brain's reward, motivation, and control systems are increasingly implicated in food addiction as an issue. Neurobiology at its center is the dysregulation of the opioid and dopamine systems, critical to pleasure and reward perception (Volkow et al. 2017; Schulte et al. 2016). The neuroadaptations caused by chronic intake of sweet, fatty, and salty foods are comparable to those observed in substance use disorders (Gearhardt and Hebebrand 2021; Morales 2022).

Obese individuals have a reduced reward response and show a greater likelihood of compulsive overeating owing to a reduction in the number of dopamine D2 receptors (Karlsson et al. 2015; Benton and Young 2016). Another illustration of the role of neurochemical pathways in food addiction is how alterations in endogenous opioid signaling are related to enhanced cravings and hedonic consumption (Karlsson et al. 2021).

According to Schulte et al. (2021), neuroimaging research has established that similar to drug addiction, food cues stimulate brain areas implicated in reward and emotion. These areas include the amygdala, orbitofrontal cortex, and anterior cingulate cortex. These neurological vulnerabilities already exist, and they are worsened by environmental factors, childhood stress, and the consumption of very processed foods (Wiss et al. 2020; Constant et al. 2020).

Traditional methods of losing weight do not fix the underlying issue of homeostatic hunger processes interacting with hedonic drives, which adds to the complexity of regulating food intake (Morales 2022; Stover et al. 2023). More accurately, neuroscience‐based treatments can be created once the neurobiology of food addiction is fully understood (Criscitelli and Avena 2016; Florio et al. 2022).

Despite increasing evidence that FA is a distinct phenotype characterized by compulsive consumption of hyperpalatable foods, the connection between FA and obesity remains contentious. Although metabolic dysregulation and energy imbalance have been the major concerns of classical obesity models for many years (Blüher 2019), FA is increasingly recognized as a behavioral disorder with alterations of the reward system and dopaminergic neuron dysfunction (Blum et al. 2017; Lindgren et al. 2018).

Neurobiological studies have established that FA is comparable to substance use disorders, particularly regarding the interruption of the dopamine and opioid systems (Karlsson et al. 2015). There must be improved diagnostic criteria to distinguish between FA and other forms of overeating. However, others believe that changes in dopamine receptors caused by obesity may be representative of adaptive behavioral reactions rather than true addiction (Benton and Young 2016). While the initial form of the Yale Food Addiction Scale (YFAS 2.0) emerged as a significant tool for diagnosing FA, its clinical applicability remains controversial because symptoms overlap with both BED and obesity in general (Carter et al. 2019).

According to Boggiano et al. (2015a, 2015b) and Florio et al. (2022), both FA and BED involve hedonic reasons for eating, but FA involves withdrawal‐like symptoms and a loss of control over highly processed foods. Due to this divergence, standard weight‐loss strategies can be less effective in individuals with FA than addiction therapies such as CBT (Howard et al. 2023). Enhancing FA detection and directing individualized treatment regimens is potentially feasible with a more evolved diagnostic paradigm that integrates behavioral, neurobiological, and psychological factors (Karnani et al. 2016). It is not possible to disentangle the effects of the food environment on FA and obesity from the individual‐level variables. Exacerbating reward system pathology and compulsive consumption are omnipresent ultra‐processed meals formulated with the aim of enhancing palatability and consumption (Fazzino et al. 2019).

Conversely, other mechanisms are also involved in systemic obesity determinants such as urbanization and socioeconomic disparity (Karnani et al. 2016). To address these two aspects, neurobehavioral and environmental, we require an overarching strategy that involves interventions at the policy level (such as food‐labeling regulations) and particular treatment modalities for FA. Longitudinal investigations must be the area of emphasis for future studies to gain more insight into the associations between FA, obesity, and comorbid mental health disorders.

### 2.1 Reward Pathways: Dopamine and Opioid System

The reward systems of the brain, particularly the dopamine‐controlled and endogenous opioid‐controlled systems, are a key part of the neurobiology of food addiction. According to Volkow et al. (2017), dopamine is vital for reinforcement learning, motivation, and reward seeking. The nucleus accumbens forms a critical component of the reward system of mesolimbic; research has shown that it can become activated by very palatable foods, especially those rich in sugar and fat (Lindgren et al. 2018).

Neuroadaptations similar to those seen in substance use have been implicated in compulsive binge eating behavior, where this pathway is repeatedly activated by food cues. The enjoyment of eating is also highly linked to opioid signaling. According to Karlsson et al. (2015), foods high in sugar and fat are more palatable and preferable due to the effects of endogenous opioids on the hedonic aspects of food intake. Obese individuals might need to eat more to compensate for not having μ‐opioid receptors in specific areas of their brain (Karlsson et al. 2015, 2021).

The opioid and dopamine pathway links intensify the reinforcement of delicious food. Moreover, when these reward systems do not function well, they can lead to “reward deficiency,” which further makes individuals seek more food in order to feel the same way (Blum et al. 2017). The same mechanisms occur with drug addiction when consumers have to take higher quantities to produce the same results they previously had. Lacking the resolution of basic neurochemical dysbalances, such neuroadaptations double back, both to reinforce inappropriate eating patterns and compound nutritional changes. Dietary structure influences these brain circuits. The brain's reward system seems particularly vulnerable to hyperpalatable ultra‐processed foods (Calcaterra et al. 2023; Fazzino 2022).

Thus, individuals consuming these foods may have exaggerated dopamine responses that result in resistant satiety signals and overeating patterns. Emerging evidence indicates that there could be therapeutic potential for the treatment of obesity and food addiction, targeting dopamine and opioid systems concurrently. Stover et al. (2023) and Carter et al. (2016) provided pharmacological methods to rehabilitate dopamine homeostasis or manipulate opioid transmission. However, further studies are required to translate these findings into effective clinical therapies.

### 2.2 Similarities to Substance Use Disorders

Both the behavioral expression and neurobiology underlying food addiction are analogous to those of more traditional substance use disorders (SUDs). As reported by Gearhardt and Hebebrand (2021) and Wiss et al. (2020), hyper‐palatable foods can induce withdrawal‐like signs, loss of control, craving, and tolerance in susceptible individuals, similar to drugs of addiction. Due to this parallel, it may be that an individual's issue with consuming too much is an addiction and not due to a lack of willpower or self‐control. There is strong evidence to support this homology in the neuroimaging literature. The amygdala, orbitofrontal cortex, and anterior cingulate cortex are brain regions that have been associated with cravings for drugs in functional magnetic resonance imaging research (Schulte et al. 2016). Similar to how individuals obtain drugs, such brain responses during food intake are possible even in the absence of physical hunger (Table 1) (Schulte et al. 2021).

**TABLE 1:** Neurobiological mechanisms linking food addiction and obesity: Focus on reward pathways and substance use disorder similarities.

Topic	Key point	Mechanism involved	References
Reward pathways	Dopamine dysregulation in obesity and BED	Reduced dopamine signaling	Milano et al. (2023)
	Psychiatric comorbidities in food addiction	Dopamine and serotonin alterations	Piccinni et al. (2021)
	Dopamine system as a common link in drug and food addiction	Dopamine motive system	Volkow et al. (2017)
	Overeating behaviors mirror drug addiction patterns	Dopamine D2 receptor downregulation	Campana et al. (2019)
	Dopamine receptors linked to behavior more than addiction	Dopamine receptor availability	Benton and Young (2016)
	Hedonic overeating explained by reward mechanisms	Opioid and dopamine pathways	Lee and Dixon (2017)
	Obesity linked to food addiction	Dopaminergic and opioid systems	Meseri et al. (2016)
	Food addiction as a component of obesity	Brain reward dysfunction	Lerma‐Cabrera et al. (2015)
	Obesity reduces μ‐opioid receptors, not dopamine D2	μ‐Opioid system	Karlsson et al. (2015)
	Restoring dopamine balance for addiction treatment	Dopamine homeostasis	Blum et al. (2017)
	Role of endocannabinoid system in food addiction	Endocannabinoid signaling	de Ceglia et al. (2021)
	Opioid‐dopamine interaction disrupted in obesity	Bariatric surgery recovery	Karlsson et al. (2021)
	Ultra‐processed foods hijack reward systems	Dopamine overactivation	Calcaterra et al. (2023)
	Diet‐induced dopamine changes	Inflammation and metabolic stress	Wallace and Fordahl (2022)
	Brain–gut–microbiome links in food addiction	Gut–brain axis	Gupta et al. (2020)
Substance use similarities	Integration of hunger and reward circuits	Homeostatic and hedonic drivers	Morales (2022)
	Neurobiology of food addiction parallels substance use	Dopamine dysregulation	Carter et al. (2016)
	Shared disruptions in reward circuits in addictions	Mesolimbic dopamine pathway	Yohn et al. (2019)
	Food addiction concept validates overeating models	Dopamine reward sensitivity	Gearhardt and Hebebrand (2021)
	Food and drug addiction share mechanisms	Dopaminergic and opioid pathways	Lindgren et al. (2018)

Patients with food addiction also have abnormalities in dopamine transmission, which are typical of substance addiction. As Benton and Young (2016) and Adams et al. (2019) found in their research, when individuals consume delicious meals too frequently, they can decrease their reward sensitivity and predispose them to overeating. The neurochemical changes observed in individuals addicted to substances such as alcohol or cocaine are replicated by these adaptations. Table 1 shows the neurobiological mechanisms linking food addiction and obesity, focusing on reward pathways and substance use disorder similarities. Similar to individuals with drug use disorders, individuals with food addiction are more likely to be impulsive, experience difficulty making decisions, and are extremely sensitive to rewards, as per behavioral and psychological studies (Criscitelli and Avena 2016; Morales 2022).

The evidence for this confluence of symptoms supports the notion that some overeating is more of an addiction than an environmental or metabolic issue. It is important to understand food addiction from a broader biopsychosocial perspective, as recent evidence has been illustrated through multidisciplinary research (Constant et al. 2020). Food addiction and substance use disorders can be promoted by an interplay between neurobiological risk factors, environmental determinants, genetic factors, and early adversities (Piccinni et al. 2021; Florio et al. 2022). By considering obesity as a complex neurobehavioral issue instead of a simple lack of self‐control, such an understanding not only directs treatment choices but also helps demystify the condition.

## 3 Highly Palatable Foods and Their Effects

Similar to substance addiction, highly palatable foods, usually rich in sugar, fat, and salt, strongly affect the brain's reward system, leading to compulsive food consumption (Gupta et al. 2020; Morales 2022). According to De Macedo et al. (2016) and Liu et al. (2016), such foods activate dopaminergic circuits in the mesolimbic system; that is, the nucleus accumbens, thus increasing hunger for more than is required by metabolism. Scientists have established that eating such meals regularly alters neural plasticity, makes us less responsive to usual signals of satisfaction, and is more prone to overeating (Morin et al. 2017; Reichelt and Rank 2017).

Furthermore, under emotional or stress‐inducing conditions, frequent intake of extremely palatable foods may result in neuroadaptive changes that enhance food‐seeking behavior (Pool et al. 2015; Jacques et al. 2019). There has been an increase in the rate of obesity and food addiction, promoted by aggressive advertisements and the wide availability of foods that are highly palatable (Fazzino 2022; Fazzino et al. 2019).

**FIGURE 1:**
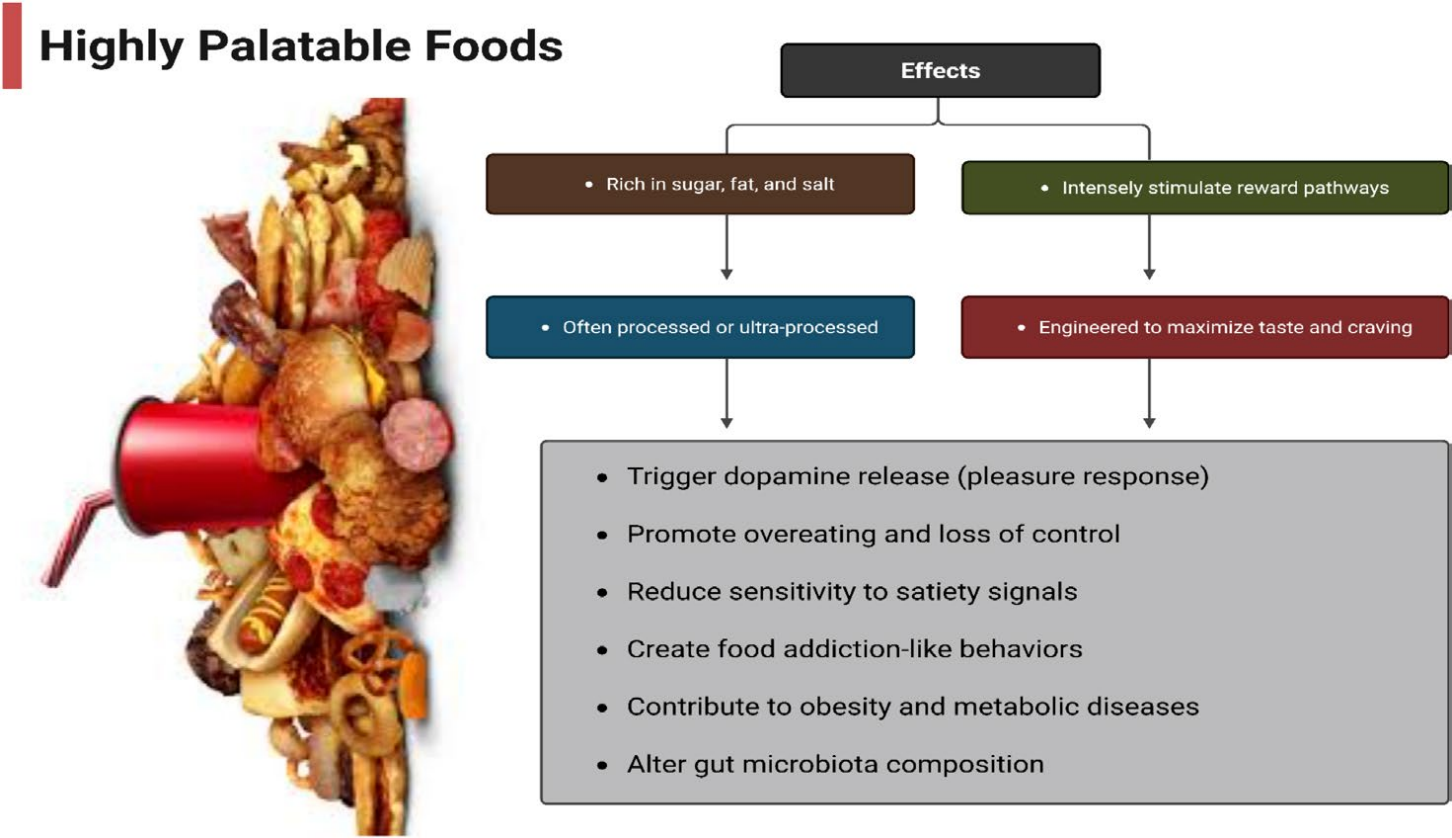
Highly palatable foods and their effects.

These foods, as indicated by Gupta et al. (2020) and Madison and Kiecolt‐Glaser (2019), not only promote the consumption of excess calories, but also disrupt the usual gut–brain signaling pathways for appetite and satiety regulation. Based on these studies, individuals struggle to manage what they eat, even if they are full, since hyper‐palatable foods hamper executive control processes, such as impulse regulation and decision‐making (Figure 1) (Morris et al. 2015; Morales 2022).

According to Schulte et al. (2020) and Vasiliu (2022), such changes in the brain and behavior due to highly palatable food require targeted interventions that extend beyond usual dietary advice; the changes in the brain and behavior induced by highly palatable food are comparable to the defining features of addiction diseases.

### 3.1 Role of Sugar, Fat, and Salt Combinations

High‐sugar, high‐fat, and high‐salt foods are more satiating because they are enjoyable (Fazzino 2022; Fazzino et al. 2019). In addition to the impact of any single macronutrient in isolation, these pairings stimulate the brain's reward system, particularly dopaminergic pathways (De Macedo et al. 2016).

Overconsumption of calories and highly palatable foods activates the mesolimbic dopamine system, which reinforces consumption behavior and promotes overconsumption (Liu et al. 2016). These food consequences are probably neurologically entrenched, as such hyperactivation is akin to that of substance use disorders (Morales 2022). As Jacques et al. (2019) maintained, the emission of dopamine to the nucleus accumbens increases following sugar intake, resulting in responses that resemble addiction.

Gupta et al. (2020) established that when bringing fats and salts together, the enjoyment experienced during consumption is enhanced, and it usually suppresses homeostatic cues of hunger. According to Morin et al. (2017), if individuals consume food rich in sugar, fat, and salt on a regular basis, the reward threshold of their brains can be adjusted; that is, they need to consume more of these foods to experience the same level of satisfaction (Afzaal et al. 2021). A neuro‐adaptive process similar to that demonstrated in chronic drug consumption underlies compulsive overeating (Morales 2022).

Moreover, as indicated by Fazzino (2022) and Gibney et al. (2017), ultra‐processed foods that contain these combinations dominate the current food environment. This results in repeated exposure, which consequently induces addictive eating behaviors. Table 2 illustrates highly palatable foods and their effects. Obesity and food addiction are widespread issues, and the availability and marketing of these products play a significant role to play here (Dutta and Haque 2021).

**TABLE 2:** Highly palatable foods and their effects.

Topic	Key point	Mechanism involved	References
Sugar, fat, salt	Palatable foods overstimulate dopamine pathways	Reward system activation	Wallace and Fordahl (2022)
Gut–brain axis	Highly palatable foods alter gut microbiota impacting behavior	Microbiota‐brain communication	Gupta et al. (2020)
Hunger regulation	Hedonic eating overrides homeostatic controls	Integration of hunger and reward systems	Morales (2022)
Industry role	US food industry promotes hyper‐palatable foods	Increased availability and reinforcement	Fazzino (2022)
Toxic diets	Overconsumption of palatable toxic diets	Metabolic and neural impairments	Dutta and Haque (2021)
Reward activation	Palatable diets hyperactivate reward circuits	Dopamine and opioid system involvement	De Macedo et al. (2016)
Food definition	Quantitative criteria for hyper‐palatable foods	Food composition analysis	Fazzino et al. (2019)
Neuronal plasticity	High‐calorie foods alter neuronal structure	Synaptic plasticity changes	Morin et al. (2017)
Stress and cues	Stress and environmental cues amplify palatable food intake	Stress‐response and reward pathways	Morris et al. (2015)
Synaptic changes	Fast synaptic remodeling after palatable food intake	VTA synaptic density increase	Liu et al. (2016)
Comfort eating	Stress‐induced preference for high‐fat, high‐sugar foods	Emotional coping via food	Pool et al. (2015)
Hedonic motives	Emotional reasons drive hyper‐palatable food consumption	Reward‐driven eating	Boggiano et al. (2015a, 2015b)
Adolescent brain	Junk food negatively impacts adolescent neurodevelopment	Reward system sensitivity	Reichelt and Rank (2017)
Sensory influence	Sensory cues beyond taste drive overeating	Sensory satiety imbalance	McCrickerd and Forde (2016)
Persistent preference	Prior exposure to methamphetamine increases preference for palatable food	Cross‐sensitization effects	Caprioli et al. (2015)
Coping and BMI	Eating tasty foods for coping correlates with higher BMI	Emotional eating reinforcement	Boggiano et al. (2015a, 2015b)
Sugar and behavior	High sugar intake impacts emotional and addictive behaviors	Stress and reward pathway alterations	Jacques et al. (2019)
Determinants of choice	Palatability, availability, and marketing affect food choice	Behavioral economics of eating	Leng et al. (2017)
Stress, diet, gut	Psychological stress affects diet and microbiota	Gut–brain–immune axis	Madison and Kiecolt‐Glaser (2019)
Pleasure and food	Hedonic thoughts promote unrestrained eating	Automatic hedonic activation	Papies et al. (2017)

According to McCrickerd and Forde (2016), a combination of ingredients makes it more difficult for the body to feel full, which could result in accidental overeating. More difficult is the fact that this biological mechanism overrides the body's natural cues, which prompt us to discontinue eating. The hedonic response created by the synergistic interaction of sugar, fat, and salt is sufficiently robust to alter regular feeding habits and play a role in the pathophysiology of FA, as indicated by Fardet and Rock (2019).

### 3.2 Impact on Eating Behavior and Satiety

According to studies conducted by Morales (2022) and Pool et al. (2015), highly palatable foods have a significant impact on eating behavior in that they enhance appeal and suppress awareness of internal satiety cues. Instead of satisfying fundamental metabolic requirements, conditioned eating through repeated exposure to these foods is the driver of consumption (Morris et al. 2015).

The body's capacity to homeostatically regulate hunger and fullness is undermined by this trend towards hedonic eating (Gupta et al. 2020). Unlike physiological signals that typically control intake, highly palatable meals trigger an overreaction from the reward systems (Morales 2022). Based on previous studies, individuals whose gut–brain communication is disrupted as a result of long‐term consumption of hyperpalatable foods have even weaker satiety mechanisms (Gupta et al. 2020). Such diets can also influence appetite regulation because of alterations in gut flora (Madison and Kiecolt‐Glaser 2019).

Furthermore, eating episodes are more prolonged and more food is used when meals are very palatable, which may lead to mindless or binge eating (Tufail et al. 2025; Fazzino 2022). As a coping mechanism that exacerbates overeating, food preference is associated with stress‐ or emotion‐induced eating (Pool et al. 2015; Boggiano et al. 2015a, 2015b). Eating such foods habitually, even when not physically hungry, makes the body react less intensely when full (McCrickerd and Forde 2016). Consequently, individuals require more food to feel full, resulting in overeating (Morin et al. 2017).

Overeating becomes a habit when very palatable foods are eaten repeatedly, which modifies neuroplasticity in the brain areas that manage motivation and reward (Liu et al. 2016). Structural and functional changes to the prefrontal cortex make it increasingly difficult to use inhibitory control over food, which in turn makes it harder to say no to delicious snacks (Reichelt and Rank 2017).

Furthermore, the obesity epidemic has been fueled by the fact that energy requirements and consumption are not connected (Morris et al. 2015). The failure of calorie‐restriction therapies that do not target the hedonic motivations for food intake is a consequence of this disconnection (Morales 2022). In summary, truly delicious food disrupts the sensitive balance between hunger, fullness, and reward, resulting in disordered eating patterns that are difficult to manage with traditional diet regimens (Gibney et al. 2017; Leng et al. 2017).

## 4 Assessment Tools and Diagnostic Criteria

The Yale Food Addiction Scale (YFAS) was constructed to operationalize eating patterns consistent with addiction‐like eating by mapping the criteria from drug use disorders to eating (Gearhardt et al. 2016). This is the key force that led to the measurement of food addiction. Large‐scale validation and broad application in clinical and non‐clinical samples have been achieved by the YFAS and its revised version (YFAS 2.0) since its introduction (Meule and Gearhardt 2019; Granero et al. 2018).

Burrows et al. (2018) and Dennis et al. (2024) showed the relationship between FA symptoms of food addiction and many psychiatric comorbidities; this test measures important attributes such as tolerance, withdrawal, and loss of control. Although the YFAS possesses numerous strengths, it is constrained by its capacity to measure the neurobiological processes underlying them because it relies largely on self‐reported behavior and subjective experience (Penzenstadler et al. 2019).

Most issues with current definitions of food addiction are due to the fact that they overlap with concepts of other disorders, including emotional eating and binge eating disorders (Hauck et al. 2020; Adams et al. 2019). Food in itself could not be the cause of the addictive reaction; instead, it could be hyper‐palatable, ultra‐processed food (Fardet and Rock 2019; Vasiliu 2022). In addition, in contrast to addictive substances, food is life‐sustaining; therefore, some wonder whether the term “addiction” is used appropriately (Wiss and Brewerton 2020).

The diagnostic process is already risky because of the stigma of addiction diagnosis and the risk of pathologizing adaptive eating (Schulte et al. 2020). The significance of integrated models that consider environmental, psychological, and biological factors in enhancing diagnostic criteria and assessment instruments is being realized as research continues (Wiss and Avena 2020).

### 4.1 Yale Food Addiction Scale (YFAS)

Translating the DSM‐IV drug use criteria to eating behaviors, the Yale Food Addiction Scale (YFAS) initially systematically measured symptoms of food addiction (Gearhardt et al. 2016). The present study aimed to evaluate the symptoms of compulsive eating, including tolerance, withdrawal, and loss of control over eating. Individuals with obesity and eating disorders belong to different groups that use the original YFAS owing to its strong psychometric properties (Meule and Gearhardt 2019).

It is clear that adjustments should be made to accommodate changes to the new DSM‐5 criteria over time. To account for such modifications, the YFAS 2.0, which has a distress/impairment scale and 11 diagnostic items, has become more sensitive and specific (Schulte and Gearhardt 2017). This version was authenticated by Granero et al. (2018) and Brunault et al. (2017) across different languages and cultures, thereby making it more universally applicable. Specifically, investigations using the Youth Food Addiction Scale (YFAS) have uniformly reported robust correlations between food addiction symptomatology and mental disorders such as anxiety, depression, and drug use disorders (Burrows et al. 2018; Dennis et al. 2024).

Additionally, as recommended by current systematic reviews, food addiction is abstemiously prevalent, rendering to YFAS criteria, especially so under the influence of binge eating disorder (Carter et al. 2019; Praxedes et al. 2022). Underreporting and misunderstanding symptoms are just a couple of biases that may affect the YFAS result because they are based on behaviors as per self‐report and are very far from flawless (Imperatori et al. 2016). However, FA remains the standard for clinical and scientific purposes of food addiction (Penzenstadler et al. 2019).

The growing incidence of the YFAS has triggered important debates about the necessity to formally recognize food addiction as a distinct mental illness (Schulte et al. 2020). Overall, this has been instrumental in advancing the field of empirical research on addictive eating habits. Aguirre et al. (2022) established that individuals who exhibit addictive‐like eating behavior, according to the Yale Food Addiction Scale (YFAS 2.0), Aguirre et al. (2022) established that individuals exhibit addictive‐like eating foods (HPFA).

Aguirre et al. (2022) identified several instances of HPFA in their concept analysis, including a model case demonstrating all 11 symptoms of substance use disorder (SUD), which supports the suggestion that HPFA may be a valid clinical condition. Conversely, there are instances where obese individuals do not engage in food addictive eating, which indicates that food addiction and obesity are two distinct but related notions (Chu et al. 2022; Aguirre et al. 2022). Due to this distinction, rather than generalized weight‐reducing interventions, individuals with HPFA require more specialized treatments, such as SUD‐based therapies.

### 4.2 Limitations in Current Definitions

Although the YFAS has significantly contributed to the study area, it has also shed light on the conceptual challenges related to the identification of food addiction. Adams et al. (2019) and Hauck et al. (2020) noted that “food” inherently differs from addictive products such as alcohol or drugs and is therefore difficult to compare with them. Preoccupations regarding redundant diagnosis are heightened by the existence of considerable common ground between food addiction as an idea and others, such as binge eating disorder and emotional eating (Burrows et al. 2017).

The specificity of the YFAS is low because it fails to separate general patterns of behavior and addiction to any specific nutrient (e.g., sugar) (Fardet and Rock 2019). Additionally, the engineered hyperpalatability of ultra‐processed foods could be a motivating force behind compulsive consumption, something that most definitions miss (Fardet and Rock 2019; Vasiliu 2022). There is also an issue with the way self‐report measures are employed frequently; these cannot possibly measure all the neurobiological features associated with addictive behaviors (Penzenstadler et al. 2019; Imperatori et al. 2016).

Food addiction has specific brain markers that need to be researched separately from substance addiction based on neuroimaging studies (Schulte et al. 2020). In addition, current definitions often confuse hedonic eating with physiological hunger, making it even more difficult to diagnose (Papies et al. 2017). Several studies have indicated that food culture and cognitive constraints, as opposed to true addiction processes, may be the origin of most “addiction‐like” symptoms (Wiss and Brewerton 2020).

Unwittingly, individuals' self‐concept and help‐seeking can be influenced by the high stigma attached to the word “addiction” (Adams et al. 2019). Therefore, we must be more aware of food psychopathology and be careful with language. Finally, of note is the fact that even though the Yale Food Addiction Scale has been effective, there must be more integrated models encompassing biological, psychological, and socio‐environmental factors for proper understanding of maladaptive eating (Wiss and Avena 2020).

## 5 Nutritional and Behavioral Interventions

Nutritional and behavioral treatments are essential to effectively treat food addiction and disordered eating behaviors. According to previous studies (Burrows et al. 2024; Kakoschke et al. 2024), consuming a diet rich in whole, minimally processed foods helps to stabilize blood sugar levels and affect the brain's reward pathways, which can subsequently decrease cravings and compulsive eating. Simons et al. (2022) discovered that whole‐food diets high in fiber, protein, and healthy fats aid in satiation and curb overconsumption. This is because processed food is highly palatable and enticing. It is important to exercise care while using diet restrictions because they can inadvertently cause vulnerable people to feel emotional or binge eating (Wiss and Avena 2020).

A superior and more sustainable solution for treating food addiction and related health conditions, such as obesity, is to comprise diet plans together with counseling. Along with lifestyle modifications, psychological interventions provide critical psychological skills, such as cognitive‐behavioral therapy (CBT) and mindful eating. According to previous research (Manasse et al. 2021; Howard et al. 2023), CBT for eating disorders assists clients in distinguishing and changing harmful thinking and behavioral schemas related to food. People can overcome emotional reactivity and food habits through mindful eating or increased sensitivity to hunger and fullness cues (Janssen et al. 2023).

Li et al. (2025) found that when CBT is combined with mindfulness interventions, it may recover treatment consequences by addressing reward‐based food behaviors as well as cognitive distortions. Moreover, new technologies, such as smartphone applications, have made behavioral therapies more accessible, providing self‐monitoring capabilities and instant support (Guluzade and Sas 2024).

The pillars of comprehensive, individualized treatment programs for food addiction and related disorders are behavioral and nutritional treatments. New insights into the neurobiology–obesity link have come from investigations of the Taq1A polymorphism of DRD2, which influences dopamine receptor density. In a meta‐analysis of 33 trials, the A1 allele of Taq1A did not invariably correlate with body mass index (BMI) in children and adults who were obese (BMI 30–40 kg/m^2^) or very obese (BMI > 40 kg/m^2^) (Benton and Young 2016).

The findings of these investigations are confounded by issues related to methodology, including the use of unrepresentative control groups or co‐occurring drug use disorders, which raise questions as to whether there is a role for the A1 allele in extreme obesity. The BMI of the general population was not significantly predicted by dopamine receptor density, as inferred from the Taq1A status. Despite the absence of a direct metabolic impact, the A1 allele could hinder weight reduction efforts owing to its association with impulsivity and compromised inhibitory control (Benton and Young 2016).

These observations question the “reward deficiency” theory of obesity and present evidence suggesting that differences in dopaminergic systems, and not the initial body mass index, dictate behavior to food interventions. In the assessment of children's food addiction (FA), The Yale Food Addiction Scale for Children (YFAS‐C) has been useful in assessing FA in children. Overweight and obese children had a higher prevalence rate of FA (19%) than community samples (12%) according to a meta‐analysis of 22 cross‐sectional studies (*N* = 6996) that set the overall FA prevalence at 15% (Xiang et al. 2023; Ahmed et al. 2025).

While FA's diagnostic comorbidity of FA with binge eating and other disordered eating behaviors requires further investigation, these findings indicate that it is a clinically meaningful construct, particularly among high‐risk individuals (Noreen et al. 2025). Since there is no longitudinal evidence regarding the long‐term consequences of FA, the association between FA severity and BMI underscores the importance of individualized therapies. Neurobiological findings identify similarities between FA and substance abuse, including alterations in dopamine signaling. However, because of the distinct expression of FA among adolescents, who often have it accompanied by emotional dysregulation, developmentally oriented assessment tools are required (Yekaninejad et al. 2021). To make a more informed decision regarding early intervention strategies, future studies should explore whether FA is a predictor of obesity trajectory or responds differently to treatment.

### 5.1 Whole Food‐Based Diets

Individuals fighting food addiction can be greatly helped by following a whole‐food diet, where a focus is placed on consuming foods that are least processed (Noreen et al. 2024). According to research conducted by Burrows et al. (2024), consuming ultra‐processed foods, which are often high in sugar, fat, and salt, can lead to eating habits like addiction due to their effect on the reward system of the brain. If you want to reduce your hyperpalatable food consumption and perhaps even your food addiction, give a whole‐food diet a try (Kakoschke et al. 2024).

**FIGURE 2:**
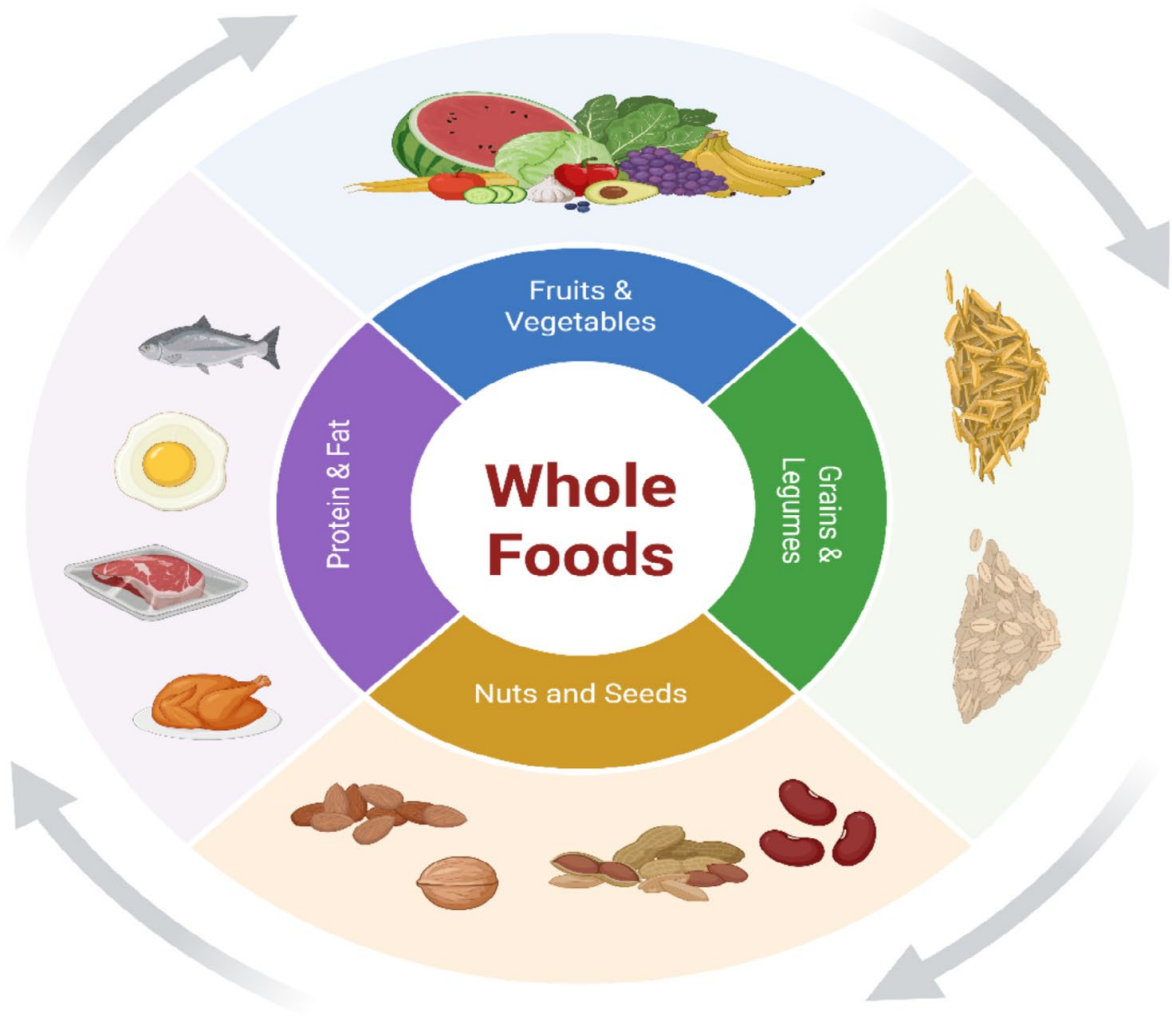
Examples of whole foods items.

Individuals who consume high amounts of fresh fruits and vegetables, lean proteins, and healthy grains maintain greater control over their hunger and have fewer cravings than those consuming high levels of processed food (Figure 2) (Simons et al. 2022). Kahathuduwa et al. (2018) also emphasized the neurological impact of food composition, where individuals on a complete meal replacement regimen were found to be more sensitive to hunger than those on an ordinary food‐based diet.

Devonport et al. (2019) also discovered that eating excessive processed food can disrupt blood sugar levels and leave individuals feeling horrible about themselves, while a diet rich in whole foods might support better mood and less emotional eating. As such, managing one's emotions is critical for avoiding harmful eating habits (Tovar Garza et al. 2021).

The role of the gut microbiota should also be taken into consideration Narmaki et al. (2022) determined that a diet rich in whole foods enhances gut health and, consequently, might influence brain function and satiety hormones, resulting in decreased binge eating. Incompletely controlled restrictive eating habits have been linked to the onset of eating disorders (Simons et al. 2022; Ruddock et al. 2019); therefore, caution should be exercised when applying such nutritional interventions. Therefore, therapy with whole foods should be flexible, satisfactory, and sustainable. Additionally, it is important to remember that food addiction and obesity are prevalent and that treating one without the other could negatively impact treatment.

Grace and Brown (2019) discovered that the best results occurred from holistic treatments that incorporated whole‐food diets and behavioral approaches. The necessity to not perpetuate the reinforcement of diet culture as well as the stigmatization of obesity sufferers has been cited in discourse pertaining to ethical discussions on how advertising extreme “clean eating” conditions as an addition to treating food addiction would pose concerns (Cassin et al. 2019). According to ElSayed et al. (2024), an overall plan for managing food addiction must involve a whole food‐based diet, which must be individualized, supportive, and part of a broader, multidisciplinary approach to promoting health.

### 5.2 Cognitive‐Behavioral Therapy (CBT) and Mindful Eating

The application of CBT in the management of compulsive overeating and other alimentary disorders has recently become more popular. The rehabilitation of addictive eating pro‐patterning is the aim of CBT for food (Manasse et al. 2021). Food addiction CBT usually aims to assist patients in regulating their emotions, urges, and cravings (Burrows et al. 2018). Behavioral treatments for food addiction, such as CBT, have been shown to reduce symptoms and normalize eating patterns (Manasse et al. 2021; Wiss and Avena 2020).

Moreover, when combined with dietary education and behavioral changes, CBT has been shown to have long‐term effects (Howard et al. 2023). In addition to mindfulness‐based treatments, mindful eating is another behavioral method that is becoming increasingly popular. Janssen et al. (2023) found that mindful‐eating individuals are less likely to engage in automatic, compulsive eating and are more likely to recognize when they are full. The biological plausibility of mindful eating as an effective intervention has been supported by recent neuroimaging findings, which revealed that it can reduce reward‐based anticipatory brain responses to food cues (Table 3) (Janssen et al. 2023). This suggests that mindful eating may also be an effective supplement to standard CBT strategies, especially for those afflicted with reward sensitivity and hedonic eating.

**TABLE 3:** Food‐based diets and cognitive‐behavioral therapy (CBT) and mindful eating.

Intervention type	Focus area	Description	Population	References
Whole food‐based diet	Dietary behavior	Emphasis on minimally processed foods to reduce addictive eating patterns	Adults with obesity	Fardet and Rock (2019)
	Craving reduction	Reduces ultra‐processed food exposure	Obese individuals	Kahathuduwa et al. (2018)
	Weight management	Meal replacement vs. whole food diets compared	Adults with obesity	Moldovan et al. (2016)
	Addiction‐like eating	Whole diets impact hedonic hunger	Adults	Taş and Gezer (2022)
	Food cue reactivity	Whole foods reduce reactivity in brain imaging	Obese adults	Kahathuduwa et al. (2018)
	Functional outcomes	Nutrient‐dense diets improve eating behavior	Obese women with food addiction	Narmaki et al. (2022)
	Eating patterns	Shift from ultra‐processed to whole foods	General population	Fardet and Rock (2019)
CBT	Psychological intervention	Targets dysfunctional thoughts related to eating	Adolescents with binge eating	Manasse et al. (2021)
	Emotional regulation	Addresses emotional triggers for eating	Adolescents	Howard et al. (2023)
	Cognitive restructuring	Reframe thoughts that trigger binge eating	BED patients	Kober and Boswell (2018)
	Eating disorders	Exposure‐enhanced CBT to manage binge eating	Adolescents	Manasse et al. (2021)
	ARFID treatment	CBT application for avoidant/restrictive food intake disorder	Children and youth	Howard et al. (2023)
	Restraint and cravings	Differentiating between restraint and addiction	Adults	Wiss and Brewerton (2020)
	Integrated approach	Combines nutrition and cognitive therapy	Eating disorder treatment	Dennis et al. (2024)
Mindful eating	Behavioral therapy	Mindful awareness during eating to manage cravings	Adults with overweight	Janssen et al. (2023)
	Reward response	Alters anticipatory food reward signals	Overweight adults	Janssen et al. (2023)
	Obesity treatment	Mindful eating apps' impact	Eating disorder users	Guluzade and Sas (2024)
	Self‐regulation	Supports autonomy in food choices	Obese adults	Chew et al. (2022)
	Hedonic thoughts	Reduce spontaneous hedonic thoughts	Restrained eaters	Papies et al. (2017)
	Craving management	Tackles reward‐driven cravings	Medical weight management patients	Kakoschke et al. (2024)

Guluzade and Sas (2024) found that mindful eating interventions using mobile applications were able to lead people with eating disorders to behavioral enhancements through real‐time reminders and tracking devices. Table 3 describes food‐based diets, CBT, and mindful eating. Technology‐based treatment can enhance access to therapy and deliver support between sessions. Although promising, CBT and mindful eating have limitations in terms of patient compliance and therapist availability. For maximum outcomes, interventions need to be customized to suit each individual's motivation, level of readiness to change, and mental profile (Chew et al. 2022).

People with more severe types of food addiction and other mental illnesses may need personalized CBT and mindfulness interventions (Kober and Boswell 2018). To further increase treatment efficiency, scientists are pondering ways to assimilate it with other neurostimulation techniques or affect‐centered therapies (e.g., transcranial direct current stimulation) (Elkfury et al. 2020). Overall, CBT and mindful eating are evidence‐based, adaptable, and complementary treatments for food addiction. Along with dietary therapies and other mobile apps, they have many prospects for personalized and comprehensive treatment regimens in the future (Grace and Brown 2019; Tovar Garza et al. 2021).

## 6 Controversies and Ethical Considerations

Regarding the risk of stigmatization and labeling, the concept of FA has also attracted immense controversy. Some feel that incorrectly labeling excessive eating as an addiction, unwholesome stereotypes, and demeaning assumptions concerning persons who have eating disorders could be strengthened. Negative psychological effects, such as social rejection and shame, might be a consequence of such labeling; Ruddock et al. (2019) write. The designation of disordered eating as a food addiction also carries the risk of oversimplifying challenging behavioral struggles and neglecting the broader psychological, social, and cultural dimensions involved. Those stigmatized as “food addicts” are more likely to be discouraged from seeking treatment since, as noted by DePierre et al. (2016), such individuals are already socially isolated along with other stigmatized individuals. When considering the moral implications of this diagnosis, one must consider, on the one hand, the advantages of accurate diagnosis and treatment versus the risks of reinforcing negative stereotypes and diminishing confidence.

Another controversial topic is the supposed role of the food industry in encouraging unhealthy eating habits and subsequently, the acceleration of food addiction‐like behavior. The primary culprits in the obesity epidemic have been identified as the food industry's marketing strategies that disproportionately target marginalized groups with high‐calorie processed foods (Browning 2017). However, most believe that food companies are responsible for the way they influence public health and that they are not. The current food system, Karnani et al. (2016), is a failure of the market because profit‐seeking interests come before considerations of public health. As it informs consumer choice and undermines efforts to encourage healthier eating, this corporate influence renders the issue of food addiction more challenging on an individual basis. The food industry's structural domination and moral obligation to protect consumers from manipulative advertising to encourage unhealthy diets should be addressed in any public health intervention that is deemed ethical (Hurlimann et al. 2017).

### 6.1 Labeling and Stigmatization

Overeating individuals might be further stigmatized and labeled as “food addicts” if this phrase is more widely used, which has ignited a heated debate. Ruddock et al. (2019) argue that the use of the term “food addicts” may inadvertently enhance the stigma related to obesity, causing shame, isolation, and poor self‐esteem. Negative psychological impacts, including enhanced despair, anxiety, and disturbed eating habits, have been attributed to this stigma, which now renders it even more damaging than ever (Westbury et al. 2023).

Researchers DePierre et al. (2016) established that the stigma surrounding the label “food addict” is equivalent to other chronic, socially stigmatized illnesses, like mental illness and alcoholism. Although food addiction is considered a multifaceted neurobehavioral illness, individuals who are labeled as “food addicts” are usually viewed as lacking in self‐control. As a result, the label tends to create harmful stereotypes and social exclusion in place of empathy. In clinical and public health settings, Cassin et al. (2019) emphasized the need to seriously consider the ethical concerns of labeling individuals. There are numerous complex explanations for obesity and eating disorders, including genetic vulnerability, environmental triggers, and psychological tension. However, validating patients' feelings and informing treatment approaches by recognizing food addiction risk oversimplifies these causes. Medicalization, in providing legitimacy and admittance to healthcare, risks pathologizing normal variability in body weight and eating behavior. Hofmann (2016) critically analyzed obesity as a socially constructed disorder. Owing to this two‐edged sword, ethical concerns have arisen about the encouragement of unhealthy and unrealistic beauty expectations that disproportionately affect women and other oppressed groups.

Policies must be transparent and frank, but also cognizant of the risk of stigmatization, as Brown (2022) noted when considering the legal effects of labeling and disclosure requirements in food marketing. If we focus too much on an individual's role in labeling, we risk scapegoating individuals for their ill health without treating systemic problems. It is important to add that labeling procedures that are imposed over addictive eating habits are often based on psychosocial adversity and trauma, as noted by Wiss et al. (2020). However, such contextual factors are rarely considered in the course of applying labeling procedures. Hence, labeling a person as a “food addict” risks victim shaming and further marginalization when they are already facing systemic injustices.

In order to avoid inadvertently reinforcing stigma, Soraghan et al. (2016) contended that “nudging” strategies for minor environmental changes, such as food labeling to promote healthier options, have to be carefully designed. Rather than shame them, social marketing should inform and empower them. Ultimately, although admitting food addiction has the potential to erode stigma towards eating disorders, consideration of ethical aspects is also required regarding labeling practices with the concern that one might look forward to disastrous consequences from various aspects, including emotion, society, and law. Multiple perspectives are necessary to develop effective policies and treatments based on thorough examinations.

### 6.2 Industry Influence and Food Marketing

No one can dispute the influence of the food industry on eating habits and the outcome of public health efforts. Browning (2017) states that the aggressive marketing strategies of the food industry, which often target vulnerable populations with unhealthy products, are a structural driver of the obesity epidemic; blaming everything on individuals is wide of the mark. According to Karnani et al. (2016), the economic goal of profit maximization in the food industry conflicts with obesity‐reducing public health strategies. They suggest that human autonomy is decreased due to the commercialization of tasty but calorie‐laden foods, as it establishes environments where self‐control is extremely difficult. Transparency in food advertising is not yet sufficient to combat business pressure, as noted by Freeman (2015), who further discusses the failures of current food labeling regulations. In the absence of strict regulatory oversight, shoppers cannot make informed choices because nutrition labels are often obscure or misleading.

Silchenko and Askegaard (2020) highlight the moralizing discourses within food advertising. They discussed how “healthy” and “unhealthy” foods are culturally associated with virtue and vice. This dualism hides the requirement that corporations be held accountable for their actions, while simultaneously upholding moral judgments regarding dietary habits and body shape. Policymakers, as Hurlimann et al. (2017) argued, must avoid paternalism and respect individuals' rights to autonomy and informed consent when addressing ethical concerns in public health interventions. To be sure, they agreed that the government must do more to counterbalance the disproportionate influence of the food industry. Most marketing strategies exploit individuals' psychological vulnerabilities.

Ad campaigns covertly construct consumerism of certain products as a morally better alternative, as evidenced by Schott (2015), through value promotion of self‐control and thinness and specifically targeting women. This aligns with and reinforces gender expectations for bodily appearance and affects how people eat. Public health communications that only discuss “food addiction” have the potential to inadvertently support industry stories that inappropriately blame people while aggressively selling dangerous products (Ruddock et al. 2019). Thus, ethical marketing reform cannot be about symptom treatment but has to address root systemic problems. Stricter regulation, more ethical advertising practices, and greater consumer education all form part of an organized initiative to rebalance the balance of power between corporate marketing and public health. Obesity and food addiction prevention programs will be seriously impaired in the absence of such measures.

## 7 Conclusion and Future Trends

Food addiction (FA) remains a controversial concept, although there is increasing evidence that it has similar neurological and behavioral characteristics to drug use disorders. The reward pathway's function at the center of the controversy is the opioid and dopamine systems, which are hijacked by artificially sweetened, high‐fat, and salty foods that trick the brain into perceiving them as full when they are not. The addictive properties of these foods render them a risk factor for metabolic dysregulation and obesity. Although the Yale Food Addiction Scale (YFAS) has been useful in diagnosing FA, the diagnostic criteria remain controversial. A particular concern is that it pathologizes normal eating habits, while others point out that it overlooks biological factors. There is hope for a decrease in compulsive eating through food and behavioral treatments, such as CBT and whole‐food diets. However, there are ethical challenges, such as the food industry's promotion of addictive foods and the stigmatization of obesity.

Public health enterprises should aim to target the regulation of food marketing and the availability of healthier options, as well as strike systemic determinants, such as people's own behaviors. To advance the understanding of FA, formulate targeted therapies, and fight societal etiologies of the obesity‐FA cycle, it is critical to develop a multidisciplinary strategy that incorporates neuroscience, psychology, and nutrition. More specific diagnostic criteria that differentiate between behavioral compulsion and physiological dependency should be the primary goal of future studies of food addiction (FA). This can be attained through biomarkers such as genetic predispositions or neuroimaging findings. The gut–brain axis and dopamine receptors are two new potential therapeutic targets that may be uncovered through neurobiological investigations. Treatment effectiveness can be improved by personalized dietary techniques to counteract individual sensitivity to reward systems. To better understand FA's comorbidity with binge eating disorder (BED) and other feeding disorders, its causality in obesity and metabolic illness, and other similar problems, longitudinal studies must be conducted.

Several social and economic determinants and the general promotion of ultra‐processed foods underlie FA, and public health actions should aim to eradicate these determinants. The prevalence of FA can be reduced through the enforcement of policy interventions such as tariffs on sugar, food labeling strictures, and restrictions on marketing directed at children. The provision of evidence‐based standards that include the amalgamation of individual therapy and prevention at the population level is undertaken by policymakers, physicians, and academics. Eventually, more empathetic management of obesity may follow from the alleviation of stigma brought about through augmented knowledge of the neurological roots of FA. Better treatments for FA and their related health effects can be achieved through the coordination of scientific, clinical, and societal initiatives.

## Author Contributions

**Sammra Maqsood:** writing – original draft (equal). **Faiyaz Ahmed:** data curation (equal). **Muhammad Tayyab Arshad:** writing – review and editing (equal). **Ali Ikram:** supervision (equal). **Muhammed Adem Abdullahi:** validation (equal).

## Disclosure

The authors have nothing to report.

## Ethics Statement

The authors have nothing to report.

## Consent

The authors have nothing to report.

## Conflicts of Interest

The authors declare no conflicts of interest.

## Data Availability

The data supporting the findings of this study are available from the corresponding author upon reasonable request.
